# Spontaneous Corneal Perforation in Ocular Rosacea

**DOI:** 10.4103/0974-9233.63070

**Published:** 2010

**Authors:** Khalid Al Arfaj, Waseem Al Zamil

**Affiliations:** Department of Ophthalmology, College of Medicine, King Faisal University, Dammam, Saudi Arabia

**Keywords:** Corneal Perforation, Doxycycline, Ocular Rosacea, Rosacea

## Abstract

Rosacea is a dermatologic condition that affects the midfacial region. Ocular rosacea is most frequently diagnosed when cutaneous signs and symptoms are also present. Ocular manifestations are essentially confined to the eyelids and ocular surface. Ocular involvement ranges from minor irritation, dryness, and blurry vision to potentially severe ocular surface disruption including corneal ulcers, vascularization and rarely perforation. We present a 49-year-old Saudi Arabian female with the diagnosis of rosacea who presented with a peripheral corneal performation. The perforation was successfully managed by surgical repair, oral doxycycline and topical steroid. The final best corrected visual acuity was 20/30 after treatment. Early referral to an ophthalmologist and careful long-term follow-up are recommended.

## INTRODUCTION

Rosacea is a common chronic skin disease of unknown etiology that primarily affects middle-aged adults.^1^–^4^ Ocular involvement has been reported in 3-58 % of patients with rosacea.^2^ Ocular manifestations range from mild blepharoconjunctivitis, to sight-threatening corneal vascularization, scaring, thinning and rarely perforation.^2^

The most common ocular presenting symptoms are foreign body sensation and burning, and the most common signs are telangiectasia and irregularity of lid margins, and meibomian gland dysfunction.^5^ Ocular rosacea is under diagnosed by ophthalmologists if the skin is not carefully examined, as the skin lesions are required to confirm the diagnosis.^2^

In this report we describe the case of a 49-year-old Saudi female who presented with typical dermatologic features of rosacea and who developed inferior corneal melting and perforation. This was managed by surgical repair of the cornea, oral doxycycline and topical steroid with good outcome.

## CASE REPORT

A 49-year-old dark- skinned Saudi female presented to our emergency room complaining of decreased vision and pain in her right eye that began 1 day prior. There was no history of trauma. She gave a past history of chronic bilateral, irritation, redness and recurrent styes which were treated by her local ophthalmologist with a lid hygiene regimen, artificial tears and topical antibiotics. The patient had a 20-year history of recurrent flushing episodes and chronic skin disease involving her face that was characterized by intermittent acne and thickening of her skin, and mild swelling of her nose. She was otherwise healthy with no history of autoimmune diseases. This was diagnosed by her dermatologist as rosacea.

Physical examination revealed eyelid thickening and nasal skin hypertrophy [Figure 1a] with surface telangectatic blood vessels on her face [Figure 1b]. Uncorrected visual acuity (UCVA) in her right eye was 20/100. Slit lamp examination revealed severe blepharitis, inferior crescent-shaped corneal melting and perforation with no infiltrates. Adjacent to the perforation, there was wedge – shaped vascularization with its base at 8 o'clock on the limbus that extended into the cornea. The Seidel test was positive indicating leakage of aqueous humor [Figure 2]. The anterior chamber was shallow and quiet. UCVA in the left eye was 20/25 and with an intraocular pressure of 17 mmHg. Slit lamp examination revealed punctuate epithelial keratopathy, without corneal neovascularization, infiltrates or thinning. The left eyelid had severe blepharitis. The crystalline lenses were clear in both eyes. The remainder of the exam was unremarkable. Laboratory workup was negative for: rheumatoid factor; serum antinuclear antibody; serum anti-neutrophil cytoplasmic antibody. Complete blood count and erythrocyte sedimentation rate were within the normal range.

**Figure 1a:**
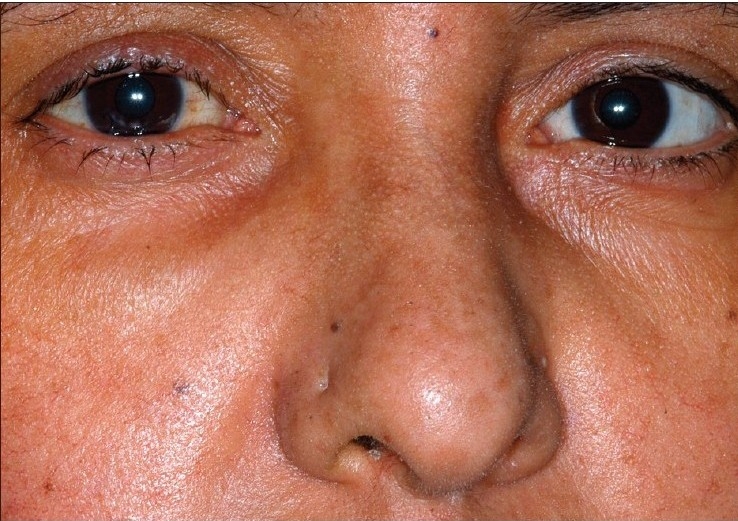
Upper eyelid shows an irregular margin and a chalazion. The malar skin shows pustules

**Figure 1b:**
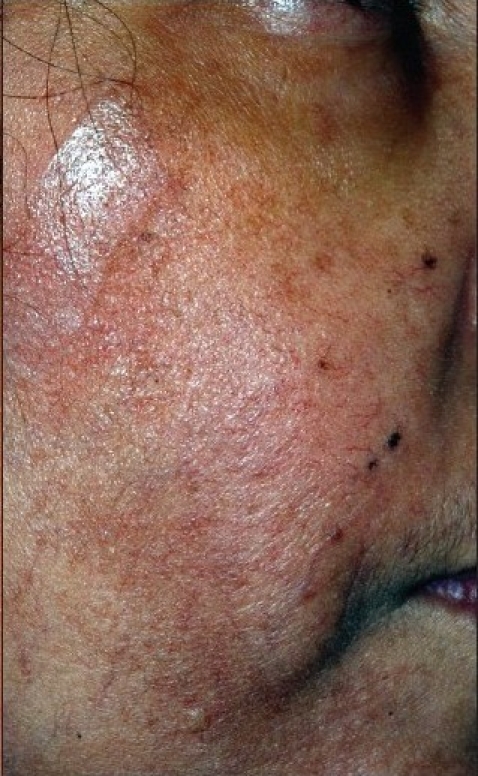
Telangectatic blood vessels on the surface of the facial skin

**Figure 2:**
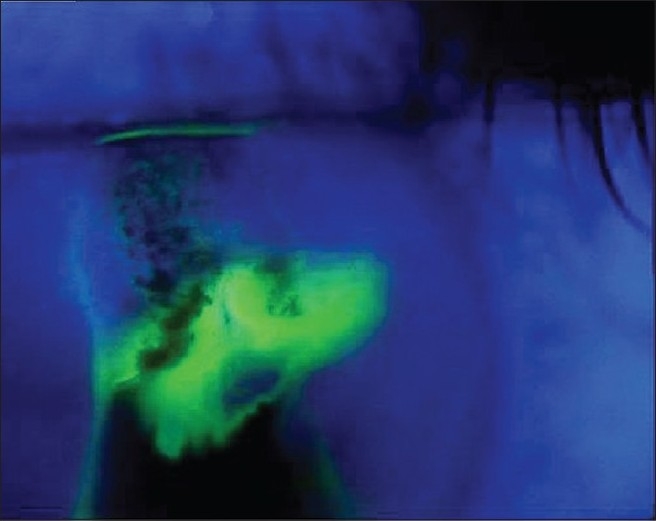
Corneal perforation involving the inferior mid-periphery with positive Seidel test

The corneal perforation in the right eye was sutured under general anesthesia. Postoperative medications included oral doxycycline 100 mg twice a day, topical steroid drops, cycloplegic agents for two months for the right eye and a lid hygiene regimen and frequent lubricants for the left eye.

**Figure 3:**
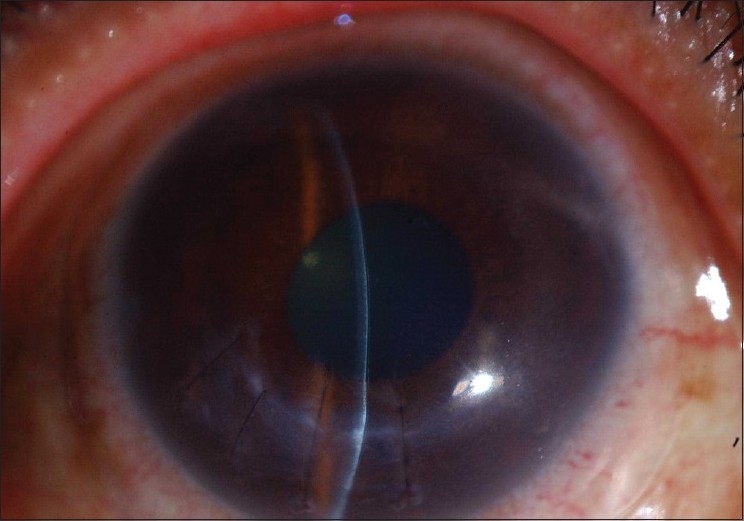
Cornea after surgical treatment for perforation

At the last follow-up visit, three months after initial presentation, the patient's best corrected visual acuity was 20/30 in the right eye, with a clear cornea centrally and a mild scar at the perforation site. The anterior chamber was quiet [Figure 3].

## DISCUSSION

Rosacea is a common chronic skin disease of unknown etiology and with a non-specific histopathological picture. It primarily affects middle-aged adults and presents with chronic erythema and telangiectasias as the site of facial flushing. The late stage of the disease is characterized by rhinophyma of the nose due to hyperplasia of the sebaceous glands.^1^,^2^ Ocular manifestations of rosecea include blepharitis, conjunctival injection, tearing, burning, recurrent chalazia, episcleritis, iritis and corneal vascularization, scarring and rarely perforation.^2^ Corneal involvement typically shows vascular invasion of the peripheral cornea with subepithelial infiltrates along the advancing vascular border. These infiltrates can progress to the central cornea, and can be accompanied by stromal ulceration or scarring.^1^

Our patient had rosacea for twenty years prior to the corneal perforation. She had inferior corneal thinning and mild vascularization adjacent to the perforation site, without corneal infiltration or ulceration. The cause for the inferior peripheral corneal thinning in rosacea was not been clearly elucidated, but might have been caused by the chronic inflammation and stromal thinning resulting from exposure to matrix degrading enzymes, such as matrix metalloproteinase 9 (MMP-9), in the inferior tear meniscus.^3^

Ocular manifestations are frequently missed in individuals with dark skin, since rosacea has traditionally been viewed as a disease affecting fair skinned individuals and pigmentation can obscure the facial skin changes. However, mild and severe ocular manifestations have been documented in black patients.^4^ We recommend that patients with darker complexion with rosacea, should be carefully evaluated to rule out ocular involvement.

A variety of surgical techniques have been used for the repair of corneal perforation due to ocular rosacea. Gracner *et al.*^6^ reported successful repair by keratoplasty of an extensive corneoscleral perforation with ocular rosacea. Jain *et al.*^7^ described the use of amniotic membrane transplantation for spontaneous corneal perforation in ocular rosacea resulting in UCVA of 20/40, 3 months postoperatively. Conjunctival flaps have also been used to manage corneal perforations and impending corneal perforations in 2 patients with acne rosacea.^8^

Our patient regained BCVA of 20/30 following simple surgical repair of the perforation and medical management with oral doxycycline, topical steroid, and cycloplegic agents without requiring an invasive procedure such as keratoplasty or amniotic membrane transplantation. In conclusion, rosacea is a chronic skin disease that commonly involves the eye and can lead to severe and vision-threatening ocular complications. Patients with dark pigmentation should also be investigated for this condition. We recommend early ocular examinations of rosacea patients. Treatment by oral doxycycline and topical steroids may result in good long term control of ocular inflammation.

## References

[1] Dursun D, Piniella AM, Pflugfelder SC (2001). Pseudokeratoconus caused by rosacea. Cornea.

[2] Akpek EK, Merchant A, Pinar V, Foster CS (1997). Ocular rosacea: Patient characteristics and follow-up. Ophthalmology.

[3] Borrie P (1953). Rosacea with special reference to its ocular manifestations. Br J Dermatol.

[4] Browning DJ, Rosenwasser G, Lugo M (1986). Ocular rosacea in blacks. Am J Ophthalmol.

[5] Barton K, Monroy DC, Nava A, Pflugfelder SC (1997). Inflammatory cytokines in the tears of patients with ocular rosacea. Ophthalmology.

[6] Gracner B, Pahor D, Gracner T (2006). Repair of an extensive corneoscleral perforation in a case of ocular rosacea with a keratoplasty. Klin Monbl Augenheilkd.

[7] Jain AK, Sukhija J (2007). Amniotic membrane transplantation in ocular rosacea. Ann Ophthalmol (Skokie).

[8] Sandinha T, Zaher SS, Roberts F, Devlin HC, Dhillon B, Ramaesh K (2006). Superior forniceal conjunctival advancement pedicles (SFCAP) in the management of acute and impending corneal perforations. Eye (Lond).

